# Laser flash photolysis study of Nb_2_O_5_/g-C_3_N_4_ heterostructures as efficient photocatalyst for molecular H_2_ evolution

**DOI:** 10.1016/j.heliyon.2023.e16772

**Published:** 2023-05-28

**Authors:** Muhammad Umair Tariq, Detlef Bahnemann, Faryal Idrees, Saman Iqbal, Fauzia Iqbal, Faheem K. Butt, Jeong Ryeol Choi, Muhammad Bilal

**Affiliations:** a Department of Physics, University of the Punjab, Lahore 54590, Pakistan; b School of Environmental Science and Engineering, Shaanxi University of Science and Technology, Xi'an 710021, Shaanxi, China; c Institut Fuer Technische Chemie, Gottfried Wilhelm Leibniz Universitaet Hannover, Callinstrasse 3, D30167, Hannover, Germany; d Laboratory of Photoactive Nanocomposite Materials, Saint‐Petersburg State University, Ulyanovskaya Str. 1, Peterhof, Saint Petersburg, 198504, Russia; e Department of Physics, Division of Science and Technology, University of Education Lahore, Township, Lahore 54770, Pakistan; f School of Electronic Engineering, Kyonggi University, Yeongtong-gu, Suwon, Gyeonggi-do 16227, Republic of Korea

**Keywords:** Nb_2_O_5_/g-C_3_N_4_, Heterostructures, Hydrogen evolution, Transient absorption spectra, Laser Flash Photolysis

## Abstract

Improvements of visible light activity, slow recombination rate, stability, and efficiency are major challenges facing photocatalyst technologies today. Utilizing heterostructures of g-C_3_N_4_ (bandgap ∼2.7eV) with Nb_2_O_5_ (bandgap ∼3.4eV) as an alternative materials for the first time, we tried to overcome such challenges in this work. Heterostructures of Nb_2_O_5_/g-C_3_N_4_ have been synthesized via hydrothermal technique. And then a time-resolved laser flash photolysis of those heterostructures has been analyzed, focusing on seeking how to improve photocatalytic efficiency for molecular hydrogen (H_2_) evolution. The transient absorption spectra and the lifetime of charge carriers at different wavelengths have been observed for Nb_2_O_5_/g-C_3_N_4_, where g-C_3_N_4_ was used for a control. The role of hole scavenger (methanol) has also been investigated for the purpose of boosting charge trapping and H_2_ evolution. The long lifetime of Nb_2_O_5_/g-C_3_N_4_ heterostructures (6.54165 μs) compared to g-C_3_N_4_ (3.1651897 μs) has successfully supported the increased H_2_ evolution of 75 mmol/h.g. An enhancement in the rate of H_2_ evolution (160 mmol/h.g) in the presence of methanol has been confirmed. This study not only deepens our understanding of the role of scavenger, but also enables a rigorous quantification of the recombination rate crucial for photocatalytic applications in relation with efficient H_2_ production.

## 1 Introduction

Photocatalysis is one of the most intriguing energy conversion processes that employ renewable and eco-friendly solar energy to generate ideal fuels and resolve the problem of photocatalytic degradation [1,2]. Hence, photocatalysis has gained a growing interest in connection with its applicability in solar energy conversion including water-splitting, photocatalytic H_2_ production, photoreduction of CO_2_, photodegradation of organic contaminants, and organic photosynthesis [[3], [4], [5], [6]]. For photocatalytic H_2_ reactions, few metal oxides have been explored but due to their large band gaps and inappropriate band potentials, they are usually not ideal for H_2_ generation or other photocatalytic applications. Some other oxides, such as CuO and Cu_2_O, have favourable band gaps but have not been preferred because of their less chemical stability and less light absorption in visible region [[7], [8], [9]]. On account of this, scientists are trying to engineer photocatalysts which are cost-effective, efficient, and chemically stable, as well as possessing suitable band potentials [9].

Graphitic carbon nitride (g-C_3_N_4_), a n-type polymer semiconductor, has earned significant importance because of its visible bandgap of 2.7eV, non-toxicity, adaptable electronic properties, and chemical stability [[10], [11], [12]]. Yet, a fast recombination rate of charge-carriers has limited its use as a bare photocatalyst. This issue has been addressed by preparing its heterojunctions with metal-oxides/metal-sulfides/metal nitrates, e.g., g-C_3_N_4_/ZnO, g-C_3_N_4_/TiO_2_, g-C_3_N_4_/WS_2_, etc. However, these heterostructures are not really ideal mainly due to expensive and complex fabrication processes [[12], [13], [14], [15]].

On the other hand, niobium pentoxide (Nb_2_O_5_) has been proven as one of the favourite materials for heterostructures preparation with g-C_3_N_4_. It belongs to n-type semiconductors with a bandgap between 3.1 and 3.4 eV, suitable for photocatalytic reactions and energy storage [15,16]. Moreover, it exhibits highly acidic properties and water-tolerant characteristics required for photoelectrochemical devices and photocatalysis. To make this material active on visible light with a high efficiency, its heterostructures such as Nb_2_O_5_/ZnO, and Nb_2_O_5_/Cu₂O [[17], [18], [19]] have been considered in previous works. Along this line, we prepared Nb_2_O_5_/g-C_3_N_4_ heterostructures which exhibit high efficiency for molecular H_2_ generation.

Herein, a hydrothermal synthetic method was used in synthesizing Nb_2_O_5_/g-C_3_N_4_ heterostructures. Compared to bare Nb_2_O_5_ and g-C_3_N_4_, the heterostructure of Nb_2_O_5_/g-C_3_N_4_ can produce high molecular H_2_, which is 75 mmol/h.g without methanol and 160 mmol/h.g with methanol. This efficient photocatalytic performance is owing to appropriate interfacial contact, which promotes the rapid photogeneration of electron-hole pairs at the Nb_2_O_5_/g-C_3_N_4_. The laser flash photolysis (LFP) technique was used as an extension to observe the trapping and lifetime of charge carriers to increase the generation of molecular H_2_. The investigation of Nb_2_O_5_/g-C_3_N_4_ LFP has not been carried out so far to the best of our knowledge.

In the LFP technique, Nb_2_O_5_/g-C_3_N_4_ was excited by a nanosecond pulsed laser for the photogeneration of active species, i.e., electron-hole pairs were monitored through transient absorption spectra. The utilization of laser pulses in this field initiated the development of transient species in flash photolysis experiments. And this is typically followed by a change in the original optical characteristics of the tested material, which was recognized by using a flash lamp. Trapping of charge carriers was observed in the presence and absence of a-holes scavenger. Trapped-hole peaks are often seen at lower wavelengths ranging from 420 to 450 nm, whereas trapped electron peaks are usually found at higher wavelengths ranging from 700 to 760 nm. However, no substantial peak could be detected on the transient spectra between 400 and 600 nm [20]. A significant increase of hole trapping was observed in the presence of a-holes scavenger, while the range of electron trapping remains the same. Additionally, a unified model equation is used to explain the decay curves of the observed transient absorption signal. Then the lifetime of Nb_2_O_5_/g-C_3_N_4_ was calculated and compared with that of g-C_3_N_4_ to show its usefulness for photocatalysts associated with the molecular H_2_ evolution.

## 2 Experimental setup

Hydrothermal synthesis was used to prepare Nb_2_O_5_ and Nb_2_O_5_/g-C_3_N_4_ heterostructures, while a conventional method was employed for g-C_3_N_4_ preparation as reported in our previous work [1].

### 2.1 Preparation of Nb_2_O_5_

Nb_2_O_5_ has been synthesized through the oxidant-peroxo route. 0.5 ml of HNO_3_ was diluted by 20 ml of deionized water, followed by adding 1g of NbCl_5_ during continuous stirring. The solution became milky white containing a few white and colourless precipitates at the bottom. Then 20 ml of H_2_O_2_ was added drop-wise which gradually changed the color of the solution to yellow, indicating the formation of niobium peroxo-complex (NPC): [Nb(O_2_)_4_]^3−^. The prepared solution was transferred into an autoclave and was kept in the oven for 24 h at 200 °C. After cooling down naturally, the prepared powder was separated through centrifugation for 30 min and thoroughly washed with deionized water. The obtained product was dried in a drying oven at 90 °C for 12 h. Afterwards, the Nb_2_O_5_ powder was annealed at 200 °C for 2 h.

### 2.2 Preparation of heterostructures of Nb_2_O_5_/g-C_3_N_4_

The preparation route of g-C_3_N_4_ has been provided in the supporting information. 0.04g of g-C_3_N_4_ was added to the prepared NPC solution for heterostructures. The obtained solution was placed in a suitable autoclave and heated for 24 h at 200 °C in an oven. After cooling down, the powder was separated from the liquid by centrifugation for 10 min, followed by drying in the oven. This approach was employed to make heterostructures with g-C_3_N_4_. The schematic illustration of the whole synthesis has been provided in the supporting information for clarification.

### 2.3 Incident photon-to-current efficiency (IPCE)

IPCE measurements can give an important insight into the contribution of photocatalysts in the conversion of incident photons into charge carriers. In this experiment, the light powder at each photo energy is measured and calculated to the IPCE value from the photocurrent according to equation (1).

(1) $$IPCE=\frac{J\times 1240}{P_{mono}\times \lambda }$$

Where J is the photocurrent density (mA·cm^−2^), $P_{mono}$ is the light power density (mW·cm^−2^) at λ. λ- wavelength of illuminating monochromatic photons.

### 2.4 Laser flash photolysis (LFP)

Nitrogen was purged on each sample for 25 min to remove air. The mixture of Nb_2_O_5_/g-C_3_N_4_ was excited by an excimer Laser (LPX200) provided by Lambda physic and the pulse duration was 20 ns. The excitation wavelength for the first sample was 680 nm and for the second sample 700 nm, whereas its laser energy per pulse was 2–10 mJ. The illumination area of the laser beam and analyzing light from the sample was 0.6 × 0.196 cm^2^. The change of reflectance was monitored through the LFP spectrometer LKS 80 from Applied Photophysics. Xenon arc lamp 150W was used as a light source. When the light was fallen on the Nb_2_O_5_/g-C_3_N_4_ sample, then the photogenerated charge carriers were produced. The reflected light was collected by the monochromator before it was sent to the photomultiplier detector (Hamamatsu R928 photomultiplier) to produce current. This change of reflectance is then recorded by the oscilloscope. The oscilloscope captured the transient with better sensitivity by adjusting the input voltage. The best part of the diffuse reflectance LFP technique is that the results obtained can be directly compared with the results of the photocatalytic experiments, although powdered samples are used in both processes. Experimental conditions for various time scales are provided in Table 1.

**Table 1:** Experimental conditions for LFP.

TOTAL TIME ACQUIRED	10 μs	20 μs
Laser Frequency	2 Hz	8 Hz
Terminal Resistance	40 Ω	5 kΩ
Excitation source (Xenon Lamp)	Pulsed	Non-pulsed
Average Shots	12 shots	200 shots
Time Resolution	100 ps/point	100 ns/point
Laser Energy Per Pulse	2–10 mJ	2–10 mJ

### 2.5 Photocatalytic molecular hydrogen (H_2_) formation

The molecular hydrogen (H_2_) evolution process was conducted using the prepared photocatalysts in the presence of a scavenger and without a scavenger. Methanol was used as a hole scavenger. In a typical experiment, 0.02 g of the prepared photocatalysts were mixed with 40 ml of deionized water. With the addition of methanol, a suspension containing 10 vol% of a-holes scavenger was obtained. The prepared solutions were degassed with Ar for 30 min and then stirred for a further 25 min. The photoreactor was then put under a solar-simulating Xenon light source (1000 W, 1.5 G) for 8 h. A homemade cooling system was used to keep the temperature constant. A gas chromatograph was used to measure the evolved H_2_ after every 1 h.

### 2.6 Material characterizations

The as-synthesized materials XRD data was recorded by a Bruker (D8 Advance) analyzer using Cu Kα (α = 0.15406 nm) radiation. The morphologies were analyzed using scanning electron microscopy (JEOL JSM-6700F, JEOL, Tokyo, Japan) equipped with a Lower Secondary Electron Image (LEI) detector. Additionally, the samples were characterised by transmission electron microscopy (TEM) using a 200 kV FEI TecnaiG2 F20 microscope. Micrographs were obtained in two modes: bright field (BF) and selected area electron diffraction (SAED). The UV–vis absorption spectrum was measured using a Varian Cary. Shimadzu 8A (Shimadzu, Kyoto, Japan) gas chromatograph equipped with a TCD detector and a 5 Å molecular sieve packed column; Ar as the carrier gas was used.

## 3 Results and discussion

### 3.1 Structural and morphological analysis

**Fig. 1:**
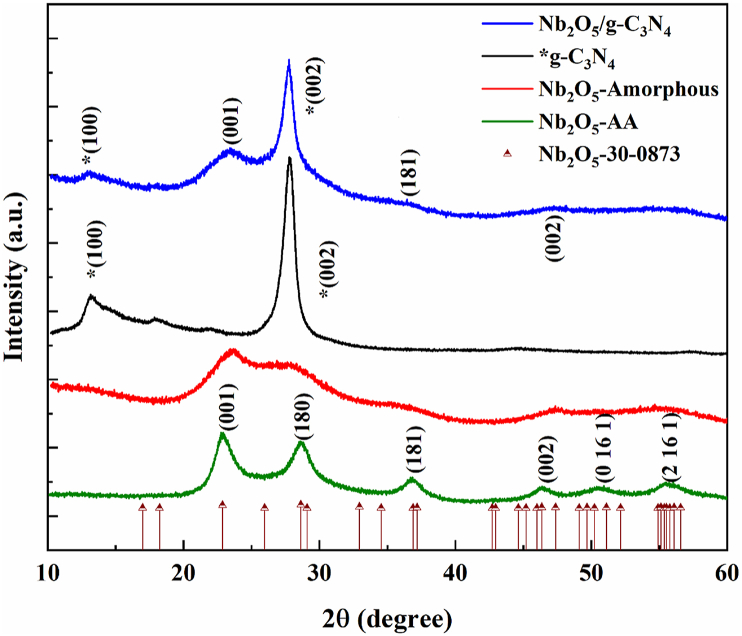
XRD patterns of g-C_3_N_4_, Nb_2_O_5_-BA, Nb_2_O_5_-AA, Nb_2_O_5_/g-C_3_N_4_ heterostructures and reference pattern of Nb_2_O_5_-30-0873.

Structural analysis was conducted by XRD for g-C_3_N_4_, Nb_2_O_5_ before annealing (BA) and after annealing (AA) and Nb_2_O_5_/g-C_3_N_4_ as shown in Fig. 1. Their corresponding Miller indices were also provided in Fig. 1. XRD measurements for Nb_2_O_5_-BA exhibited a combination of niobic acid and amorphous Nb_2_O_5_, therefore, slight distortion from the original crystalline structure was observed. While after annealing at 200 °C, the peaks were well-matched with Nb_2_O_5_ orthorhombic phase (T-Nb_2_O_5_): ICCDS#30–0873 (a = 6.17 Å, b = 29.97 Å and c = 3.93 Å). The lattice constants were calculated by using equation (2) and were found in good agreement with the T-Nb_2_O_5_ (a = 5.96 Å, b = 29.26 Å and c = 3.89 Å) except ‘a’ was found smaller. This change possibly be due to changed morphology.

(2) $$\frac{1}{d^{2}}=\frac{h^{2}}{a^{2}}+\frac{k^{2}}{b^{2}}+\frac{l^{2}}{c^{2}}$$

XRD of g-C_3_N_4_ shows two peaks observed at 13.25° and 27.8° that can be indexed as (100) and (002), respectively. The (100) diffraction plane corresponds to the characteristic interplanar system and the (002) plane corresponds to inter-layer structural packing. XRD pattern of Nb_2_O_5_ exhibited mild and broad diffraction peaks indicating its amorphous behaviour. In comparison to pure Nb_2_O_5_ and g-C_3_N_4_, Nb_2_O_5_/g-C_3_N_4_ composites showed a combination of both characteristic peaks. High characteristic peak of g-C_3_N_4_ (002), suggesting a major pairing between g-C_3_N_4_ and Nb_2_O_5_. Energy-dispersive X-ray spectroscopy (EDX) analysis of the Nb_2_O_5_/g-C_3_N_4_ heterostructures is given in Figure S1 (a-b) of the supplementary material, which confirmed the existence of the elements Nb, C, O, and N for the prepared sample. Moreover, it confirmed the Nb_2_O_5_ formation for amorphous. EDX measurements revealed that Nb_2_O_5_ agglomerates interact strongly with g-C_3_N_4_.

**Fig. 2:**
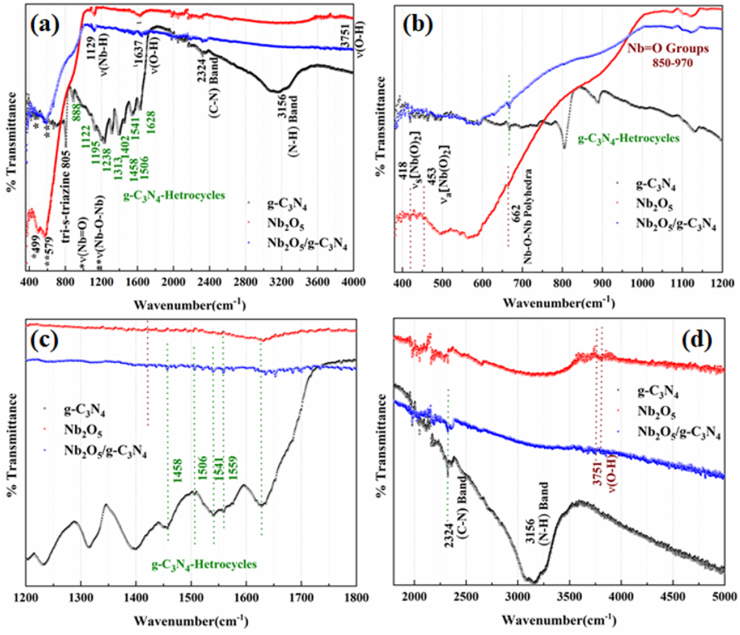
FTIR spectra of g-C_3_N_4_, Nb_2_O_5_ and Nb_2_O_5_/g-C_3_N_4_ heterostructures (a) Full Scan, (b) 400-1200 cm^−1^, (c) 1200-1800 cm^−1^ and (d) 1800-5000 cm^−1^.

The FTIR spectra of g-C_3_N_4_, Nb_2_O_5_ and Nb_2_O_5_/g-C_3_N_4_ were shown in Fig. 2. The spectrum of pure g-C_3_N_4_ presented all bands described in the literature [[21], [22], [23]] with intensive bands at 808 cm^−1^, 886 cm^−1^ and in the range of 1122–1632 cm^−1^, which are assigned to out-of-plane bending vibration of heptazine rings, deformation mode of N–H bonds and stretching modes of C–N heterocycles, respectively. These are also known as g-C_3_N_4_ heterocycles which has been marked in Fig. 2(a). The spectrum of pure Nb_2_O_5_ presented the symmetric stretching mode of Nb–O polyhedral (NbO_6_, NbO_7_, and NbO_8_), stretching of Nb=O groups, symmetric stretching ν_s_[Nb(O)_2_] and the asymmetric stretching ν_a_[Nb(O)_2_], indicating the presence of a small amount of coordinated peroxide on the Nb(V) ions [1]. The vibration of OH groups v(O–H) of adsorbed water was also observed. Details are provided in Table 2. The FTIR spectra of the Nb_2_O_5_/g-C_3_N_4_ heterostructures exhibited characteristic peaks of both phases as marked in Fig. 2(b–d) (see Table 3).

**Table 2:** FTIR Vibrational Data of Nb_2_O_5_ and g-C_3_N_4_ [1,[21], [22], [23]].

*Nb*_*2*_*O*_*5*_	418 cm^−1^	453 cm^−1^	499 cm^−1^	579 cm^−1^	850-970 cm^−1^	3751 cm^−1^
	ν_s_[Nb(O)_2_]	ν_a_[Nb(O)_2_]	ν(Nb=O)	ν(Nb–*O*–Nb)	ν(Nb=O)	ν(O–H)
*g-C*_*3*_*N*_*4*_	805 cm^−1^	886, 1122-1632 cm^−1^		2324 cm^−1^		3156 cm^−1^
	Tri-s-triazine	g-C_3_N_4-_heterocycles		C–N		N–H

**Table 3:** Results of nonlinear second-order kinetic fit at 680 and 720 nm for Nb_2_O_5_/g-C_3_N_4_ after excitation with 2–10 mJ/cm^2^.

Parameters	Wavelength 680 nm	Wavelength 720 nm
A	1.42663 × 10^44^ ± 1.63283 × 10^45^	1.01592 × 10^45^ ± 2.61135 × 10^45^
B	4.05706 ± 0.17132	0.64582 ± 0.01425
K	−9.84014 × 10^13^ ± 6.76485 × 10^19^	2.7570910^22^ ± --
T	−3.39871 × 10^14^ ± 2.34827 × 10^20^	2.6864 × 10^25^ ± --

**Fig. 3:**
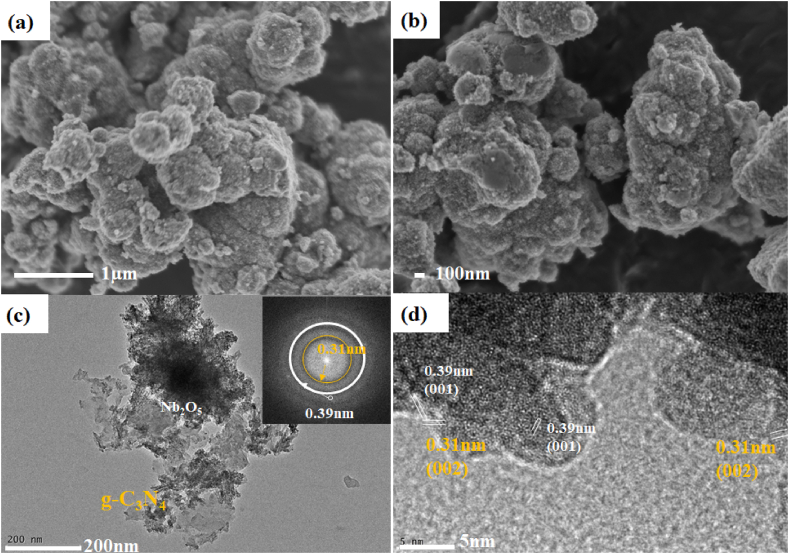
SEM images of (a) Nb_2_O_5_ and (b) Nb_2_O_5_/g-C_3_N_4_, (c) TEM image (inset SAED patterns) and (d) HRTEM image of Nb_2_O_5_/g-C_3_N_4_ heterostructures.

SEM images of as-synthesized Nb_2_O_5_ and the Nb_2_O_5_/g-C_3_N_4_ heterostructures are presented in Fig. 3(a–b), respectively. The SEM image of g-C_3_N_4_ is provided in Fig. S2 of the supporting information. The hierarchical nanospheres of Nb_2_O_5_ and Nb_2_O_5_/g-C_3_N_4_ heterostructures could be observed. A TEM analysis of Nb_2_O_5_/g-C_3_N_4_ was performed as shown in Fig. 3(c–d). Lattice fringes were found for Nb_2_O_5_ and g-C_3_N_4_ as shown in the HRTEM image of Fig. 3 (d). SAED pattern has also been provided in Fig. 3 (c-inset). The BET surface areas of Nb_2_O_5_, g-C_3_N_4_, Nb_2_O_5_/g-C_3_N_4_ before annealing and Nb_2_O_5_/g-C_3_N_4_ after annealing at 200 °C have been provided in Table S1. The specific surface area (SSA) decreased after heterostructure formation with g-C_3_N_4_. Annealing processes further reduce the SSA which indicates a decrease in the surface defects.

### 3.2 UV–vis and IPCE spectroscopy

**Fig. 4:**
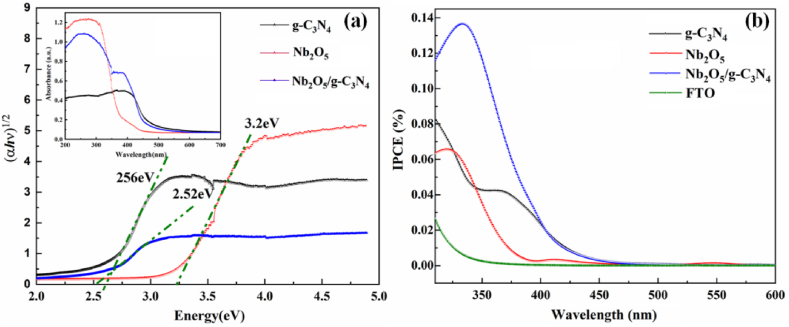
(a) Tauc's plots for Nb_2_O_5_, g-C_3_N_4_, and g-C_3_N_4_/Nb_2_O_5_ heterostructures obtained through UV–Vis-diffuse Absorption curves provided inset, (b) IPCE spectra of g-C_3_N_4_, bare Nb_2_O_5_, Nb_2_O_5_/g-C_3_N_4_ and FTO in 0.2 M NaSO_4_ electrolyte without any sacrificial reagents conducted with a standard two electrodes in a short circuit.

Bandgap energies and corresponding absorption spectra of g-C_3_N_4_, Nb_2_O_5_, and Nb_2_O_5_/g-C_3_N_4_ were shown in Fig. 4. The bandgap energies were estimated by the absorption coefficient (inset) of Tauc's plot as shown in Fig. 4 (a). g-C_3_N_4_ and Nb_2_O_5_ possess indirect transition band gaps, *i.e.,* 2.56 eV and 3.1 eV, respectively. In addition, the bandgap of Nb_2_O_5_/g-C_3_N_4_ heterostructures was red-shifted to 2.52 eV. The observed band gap helped to increase the photocatalytic efficiency of the prepared heterostructures.

IPCE (Incident Photon to Current Conversion Efficiency) spectra of as-prepared electrodes were measured in electrolyte without any sacrificial reagents and conducted with a standard two electrodes in a short circuit. The corresponding IPCE spectra of bare Nb_2_O_5_, g-C_3_N_4_, Nb_2_O_5_/g-C_3_N_4_ and FTO are presented in Fig. 4 (b). Nb_2_O_5_/g-C_3_N_4_ exhibited the highest IPCE values along the scanned wavelength in the UV region in comparison to Nb_2_O_5_ and g-C_3_N_4_. The reduced recombination rate of photoinduced electrons and holes provides a meaningful contribution to an increment of IPCE for the Nb_2_O_5_/g-C_3_N_4_ electrode. Its photoactivity is also observed in the visible light region from 400 nm to 450 nm, which IPCE value is higher than that of Nb_2_O_5_. These results indicated that the introduction of g-C_3_N_4_ not only highly enhances the photoelectric conversion ability of Nb_2_O_5_/g-C_3_N_4_ in the UV light region, but also extends its light capture into the visible light region.

### 3.3 Charge carriers trapping in Nb_2_O_5_/g-C_3_N_4_

**Fig. 5:**
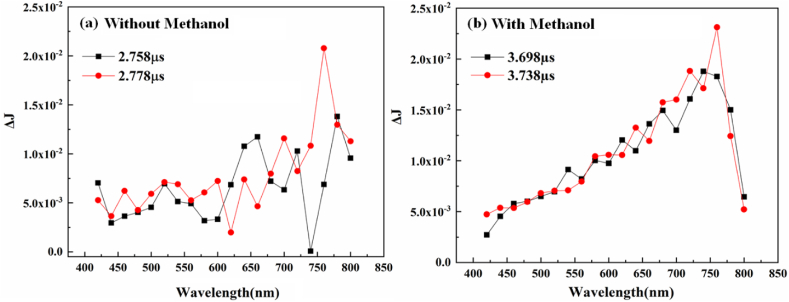
Transient absorption spectra of the Nb_2_O_5_/g-C_3_N_4_ composite were measured at different times for wavelengths ranging from 400 to 850 nm (λ_ex_ = 320 nm) (a) without methanol and (b) with methanol.

Nb_2_O_5_/g-C_3_N_4_ bandgap is 2.52 eV and by IPCE spectra the maximum was observed at 350 nm, therefore, the irradiated laser pulse of λ_ex_ = 320 nm was used. The transient absorption spectra of Nb_2_O_5_/g-C_3_N_4_ were observed for different wavelengths ranging between 400 and 800 nm against ΔJ. The experiment was performed under inert conditions (N_2_ environment) and the recombination of pure charge carrier kinetics was observed with and without a hole-scavenger (methanol). After a full spectrum scan ranging from 400 to 850 nm two different values of time was used to investigate charges in the transient spectra, as shown in Fig. 5. Without methanol, the transient absorption has shown a random pattern as shown in Fig. 5(a). With methanol, the transient absorption strongly increases at 680 nm and 720 nm, while decreasing at 425 nm as shown in Fig. 5 (b). This strategy employed that the trapped holes have transient absorption peaks around 420–450 nm and peaks of trapped electrons ranging from 700 to 760 nm at higher wavelengths. Therefore, we further observed the transient absorption spectra at 680 nm and 720 nm to calculate the lifetime of electrons which are possibly taking part in the increased photocatalytic efficiency for H_2_ generation.

### 3.4 Transient absorption kinetics and second-order fit on transient signals

To observe the transients in the oscilloscope, the Beer-Lambert law was applied by using equation (3). Herein, photogenerated species were detected by the fraction of transmitted light.

(3) $$A=-\log\left(\frac{I_{t}}{I_{0}}\right)$$

**Fig. 6:**
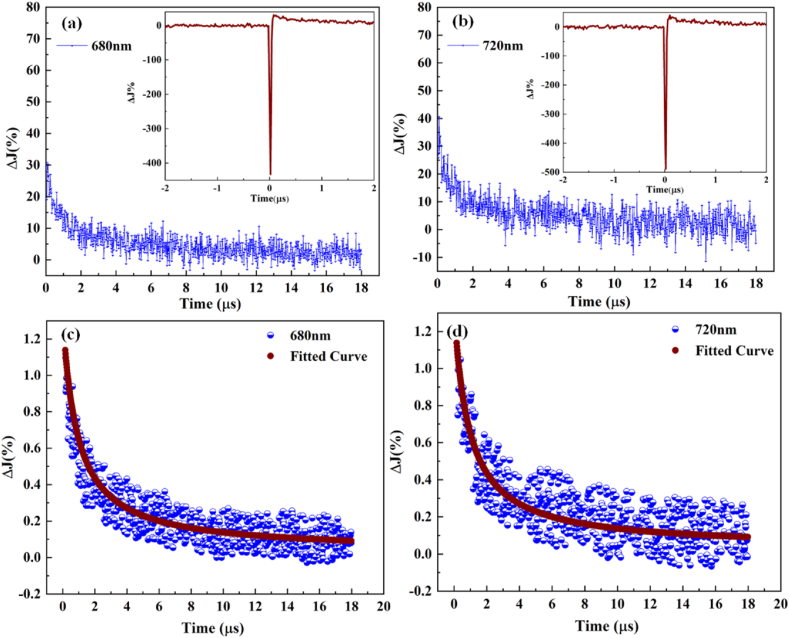
Reflectance transition of Nb_2_O_5_/g-C_3_N_4_ at (a) 680 nm and (b) 700 nm after laser excitation (λ_ex_ = 320 nm); Modified percentage reflectance of Nb_2_O_5_/g-C_3_N_4_ after excitation with 2–10 mJ/cm^2^ at (c) 680 nm and (d) 720 nm and laser excitation (λ_ex_ = 320 nm).

Where $I_{0}$ is the incident intensity and $I_{t}$ is the fraction of light measured after interaction with the powder sample. Fig. 6 represents the transient absorption kinetics at (a) 680 nm and (b) 700 nm in an N_2_-methanol atmosphere for Nb_2_O_5_/g-C_3_N_4_ after 0.078 μs in the 10 μs measurements. The full transient signal can be seen in the inset of Fig. 6(a–b). In the beginning, there is a negative peak associated with scattering from UV lasers, which was used as the source of excitation. The 40 Ω terminal resistance enables a short period of rising and allows the signal to be easily detected after 0.078 μs. The 5 kΩ high resistance used for the 20 μs measurements eventually led to a much longer detector rise time. Before kinetic analysis, the first 0.08 μs of observations were removed as they did not reflect the transient signal.

The data point was reduced to 897 for the easy processing of the transient signal by removing 103 points. The sample reflecting changes was determined by equation (4).

(4) $$\Delta J=1-10^{-Abs}=\frac{I_{0}-I}{I_{0}}$$

As predicted that LFP was used to observe the recombination of charge carriers corresponding to second-order kinetics [22]. Many scientists worked on this kinetics, but Tamaki et al. [23] are the ones who provided the detailed kinetic analysis regarding second-order processes. However, they focused only on signals ranging from picosecond (ps) to nanosecond (ns). In the present work, a second-order kinetic fit was used, and the signal ranges have been taken from μs-ns. A second-order fit function that we have used is provided in equation (5) [24]. The fitted model has been shown in Fig. 6(c–d).

(5) $$Y=\frac{t\times A}{k\times A\times X+1}+B$$

Where Y is the second-order adsorption, *A* is the altitude at which transient signals are detected, *k* is the recombination constant, *B* is the residual signal after fitting, X is the varying parameter used for fitting and t is the time taken by the curve of decay (μs-fs). By using the above equation, charge carrier signals were fitted with the obtained parameters by taking three different percentage change values (ΔJ %) at 680 nm and 720 nm. This model explains the enhanced performance of the composite material in photocatalytic hydrogen production experiments.

### 3.5 Calculation for lifetime

Table 4 presents the comparison of the lifetimes of g-C_3_N_4_ and Nb_2_O_5_/g-C_3_N_4._ To calculate the average lifetime of Nb_2_O_5_/g-C_3_N_4_, we have calculated the lifetime of g-C_3_N_4_. After plotting the values, we plotted a graph between these values and then integrated this graph by clicking on the analysis, mathematics and then integrated the function. After performing all these steps correctly, we finally generated the area A_1_ = 3.79384980 × 10^−10^ which was taken in the numerator of equation (5). Then we plotted another graph by doing the same process as mentioned above and calculated the area A_2_ = 1.1986165 × 10^−06^ which was taken in the denominator of equation (5). By dividing A_1_ by A_2_, the average lifetime of the decay curve was calculated as 3.1651897 μs. Similarly, the average lifetime of Nb_2_O_5_/g-C_3_N_4_ was calculated as 6.54165μs by using the A_1_ = 3.88509 × 10^−13^, A_2_ = 5.939 × 10^−08^.

**Table 4:** Comparison of lifetimes of g-C_3_N_4_ and Nb_2_O_5_/g-C_3_N_4_.

Material	A_1_	A_2_	Average Lifetime
g-C_3_N_4_	3.79384980 × 10 ^−10^	1.1986165 × 10 ^−06^	3.1651897 μs
Nb_2_O_5_/g-C_3_N_4_	3.88509 × 10^−13^	5.939 × 10 ^−08^	6.54165 μs

### 3.6 Photocatalytic evolution of molecular H_2_

**Fig. 7:**
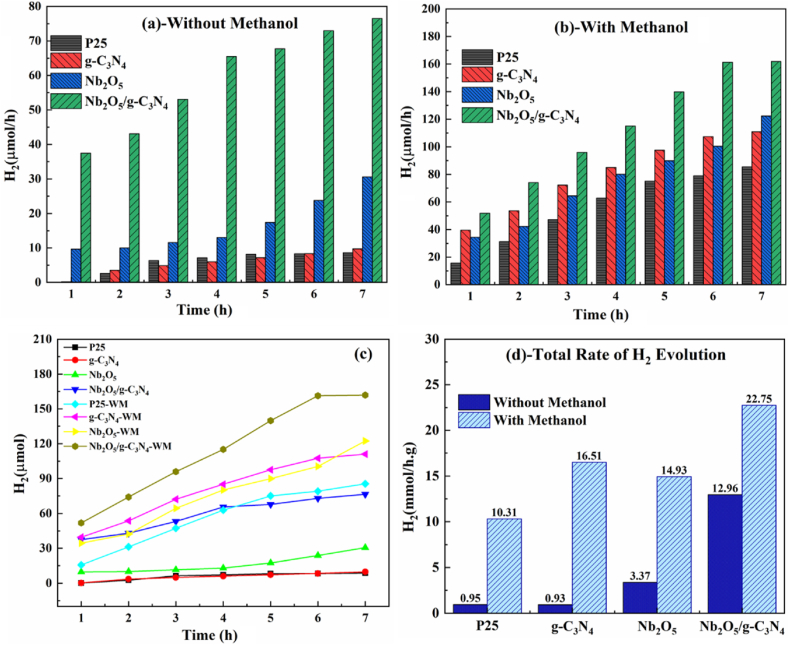
Molecular H_2_ evolution for P25, Nb_2_O_5_, g-C_3_N_4_, and Nb_2_O_5_/g-C_3_N_4_: (a) without methanol, (b) with methanol, (c) overall comparison, and (d) total rate with and without methanol.

Detailed research was conducted to determine the effect of various factors on the rate of molecular hydrogen evolution. Initially, photocatalytic production of molecular H_2_ for P25, g-C_3_N_4_, Nb_2_O_5_, and Nb_2_O_5_/g-C_3_N_4_ was performed with and without methanol. Fig. 7(a–b) exhibits the evolved H_2_, while Fig. 7(c–d) the overall comparison and total rate of H_2_ evolution with and without methanol. Heterostructures prepared with and without methanol show a higher molecular H_2_ than P25, g-C_3_N_4_, and Nb_2_O_5_. An increase in the H_2_ production rate in the presence of methanol is evidence of efficient interfacial charge separation. Nb_2_O_5_/g-C_3_N_4_ heterostructures generated the highest hydrogen production rate (22.75 mmol/h g) due to their wide surface area and suitable band positions. Thus, Nb_2_O_5_/g-C_3_N_4_ heterostructures with enhanced photocatalytic activity were produced by loading g-C_3_N_4_ as an electron-hole separator. The current results of g-C_3_N_4_, Nb_2_O_5_, and Nb_2_O_5_/g-C_3_N_4_ were also compared with previous reports [13,[21], [22], [23], [24]]. Our results showed a higher molecular H_2_ rate, even without methanol.

### 3.7 Photocatalytic mechanism of g-C_3_N_4_/Nb_2_O_5_

**Fig. 8:**
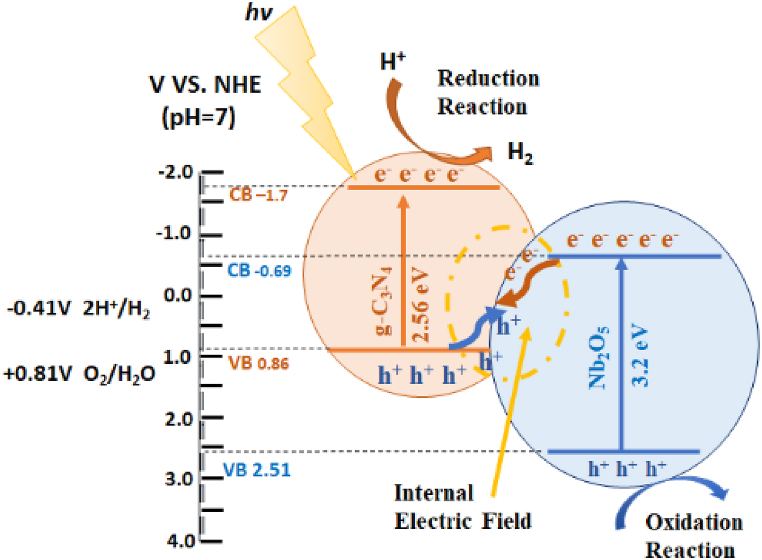
Schematic illustration of the associated photocatalytic mechanism.

Direct Z-scheme charge transfer favoured the band positions of Nb_2_O_5_ and g-C_3_N_4_. As shown in Fig. 8, under solar light electrons on the surface of the Nb_2_O_5_ conduction band (CB) moved to the g-C_3_N_4_ valence band (VB) and then recombined with holes. Electrons on the CB of g-C_3_N_4_ react with H^+^ to generate molecular H_2_. The holes on the VB of Nb_2_O_5_ can undergo possible oxidation reactions. Compared to pure g-C_3_N_4_, the Nb_2_O_5_/g-C_3_N_4_ heterostructures exhibited higher photocatalytic activity for molecular hydrogen production due to the longer lifetime of photogenerated charges. These results indicated the generation of efficient heterojunctions between the g-C_3_N_4_ and Nb_2_O_5_ phases, which promoted charge carrier transfer and enhanced the lifetimes of the photogenerated charges. The proposed Z-Scheme photocatalysis mechanism has been provided in Fig. 8. The calculated band edges by Mott-Schottky analysis have been provided in Fig. S4.

## 4 Conclusions

Hydrothermal synthesis was employed to produce Z-scheme Nb_2_O_5_/g-C_3_N_4_ heterostructures, which reported the high molecular H_2_ evolution with and without a hole scavenger as compared to pure g-C_3_N_4_ and Nb_2_O_5_. These heterostructures exhibited higher photocatalytic activity due to the longer lifetime of photogenerated charge carriers, where the lifetime was calculated through the Laser Transient Absorption Spectra. This study was conducted first time for Nb_2_O_5_/g–C_3_N_4_–prepared heterostructures.

The contribution of hole scavenger for possible charge trapping and increased H_2_ evolution was demonstrated. The laser flash photolysis study in this work showed that the hole and electron trapping occur in the wavelength ranges ∼440 nm and ∼750 nm in the absence of methanol. But, in the presence of methanol, hole trapping strongly increased whereas electron trapping remains the same. A second-order kinetic fit was applied to describe the recombination of charge carriers. Our model well predicts the charge carrier signal in Nb_2_O_5_/g-C_3_N_4_ that was seen in the nanosecond to microsecond time scale after being excited by a laser flash. Meanwhile average lifetimes of both g-C_3_N_4_ (∼3.1651897 μs) and Nb_2_O_5_/g-C_3_N_4_ (∼6.54165 μs) were shown, we observed a higher rate of molecular H_2_ evolution for Nb_2_O_5_/g-C_3_N_4_ heterostructures.

## Author contribution statement

Muhammad Umair Tariq: Analyzed and interpreted the data; Wrote the paper.

Detlef Bahnemann: Conceived and designed the experiments; Contributed reagents, materials, analysis tools or data.

Faryal Idrees: Conceived and designed the experiments; Performed the experiments; Wrote the paper.

Saman Iqbal: Performed the experiments; Wrote the paper.

Fauzia Iqbal: Contributed reagents, materials, analysis tools or data; Wrote the paper.

Faheem K Butt: Performed the experiments; Analyzed and interpreted the data.

Jeong Ryeol Choi: Contributed reagents, materials, analysis tools or data.

Muhammad Bilal: Analyzed and interpreted the data.

## Data availability statement

Data included in article/supp. material/referenced in article.

## Funding

This work was supported by the National Research Foundation of Korea(NRF) grant funded by the Korea government (MSIT) (No.:NRF-2021R1F1A1062849). This study was supported by Alexander Von Humboldt, Germany, Saint Petersburg State University, Russia and Pakistan Science Foundation (PSF-NSFC-IV/Phy/P-PU(31)).

## Declaration of competing interest

The authors declare that they have no known competing financial interests or personal relationships that could have appeared to influence the work reported in this paper.
