# Supplementary material for: Laser flash photolysis study of Nb2O5/g-C3N4 heterostructures as efficient photocatalyst for molecular H2 evolution
Source: Heliyon. 2023 May 28;9(6):e16772. doi: 10.1016/j.heliyon.2023.e16772 (PMC10248273; doi:10.1016/j.heliyon.2023.e16772)
Supplement: Multimedia component 1 [file mmc1.docx]

### Supporting Information

# **Laser Flash Photolysis Study of Nb_2_O_5_/g-C_3_N_4_ Heterostructures as Efficient Photocatalyst for molecular H_2_ Evolution**

Energy-dispersive X-ray spectroscopy (EDX) analysis of the Nb_2_O_5_/g-C_3_N_4_ heterostructure is given in Figure S1. (a-b), which acknowledged the existence of the elements Nb, C, N, and O for the prepared sample. The EDX results confirm that agglomerates comprised of Nb_2_O_5_ NPs are in intimate interaction with the g-C_3_N_4_.


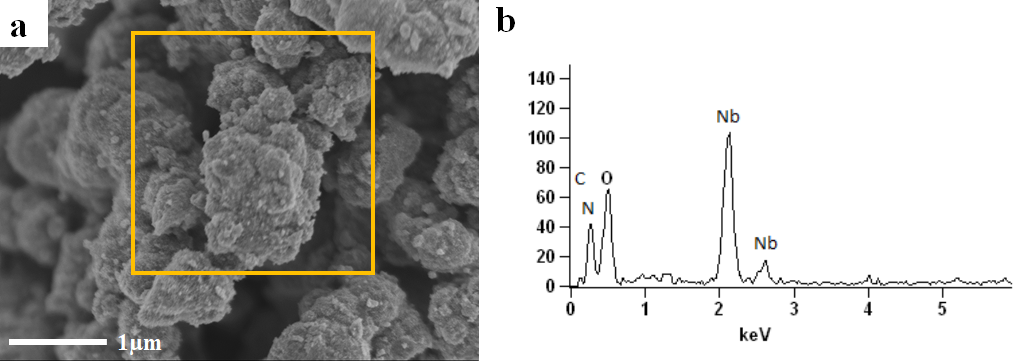


**Figure S1.** EDX analysis of (a) selected region and (b) mapping for the selected area.

**Table S1.** Specific surface area of Nb_2_O_5_, g-C_3_N_4_, Nb_2_O_5_/g-C_3_N_4_ before annealing and Nb_2_O_5_/g-C_3_N_4_ after annealing at 200^o^C.

| **Calcination Temp.** | **Surface Area (m^2^g^-1^)** |
| --- | --- |
| **Nb_2_O_5_** | **259.12** |
| **g-C_3_N_4_** | **9** |
| **Nb_2_O_5_/g-C_3_N_4_**-**BA** | **249.26** |
| **Nb_2_O_5_/g-C_3_N_4_**-**AA** | **236.86** |

**Preparation of g-C_3_N_4_**

10 g of melamine were placed in a crucible with a cover lid on top and then annealed at 550 ^0^C for 3 h in a muffle furnace employing a heating rate of 10 ^0^C min^-^1. After natural cooling, a yellow powder of bulk g-C_3_N_4_ was obtained.


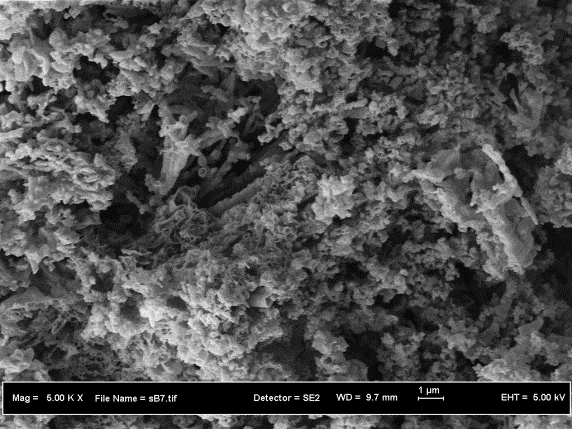


**Figure S2.** FESEM image of g-C_3_N_4_

**
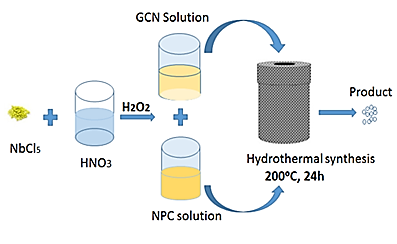
**

**Figure S3.** Schematic representation of Nb_2_O_5_ and Nb_2_O_5_/g-C_3_N_4_ synthesis.

**Photoelectrochemical Conversions**

$V_{CB}\approx V_{FB\left( NHE, pH 7 \right)}=V_{FB\left( \frac{Ag}{AgCl}, pH 5.6 \right)}-0.059\left( 7-5.6 \right)$ **Equation S1**

$V_{VB}= V_{CB}+ \frac{E_{g}}{e}$ **Equation S2**


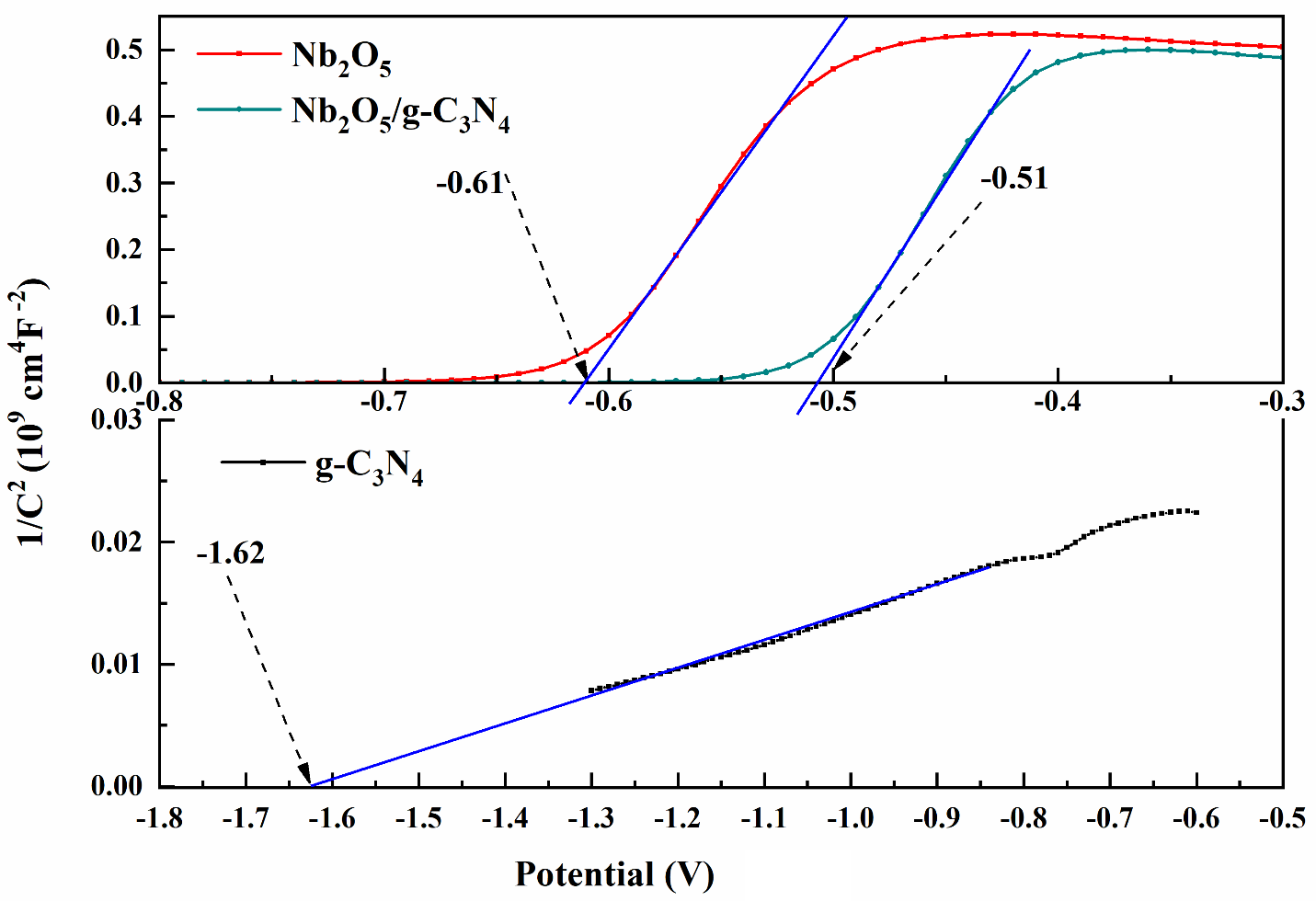


**Figure S4.** Mott-Schottky plot of g-C_3_N_4_, Nb_2_O_5_ and Nb_2_O_5_/g-C_3_N_4_.


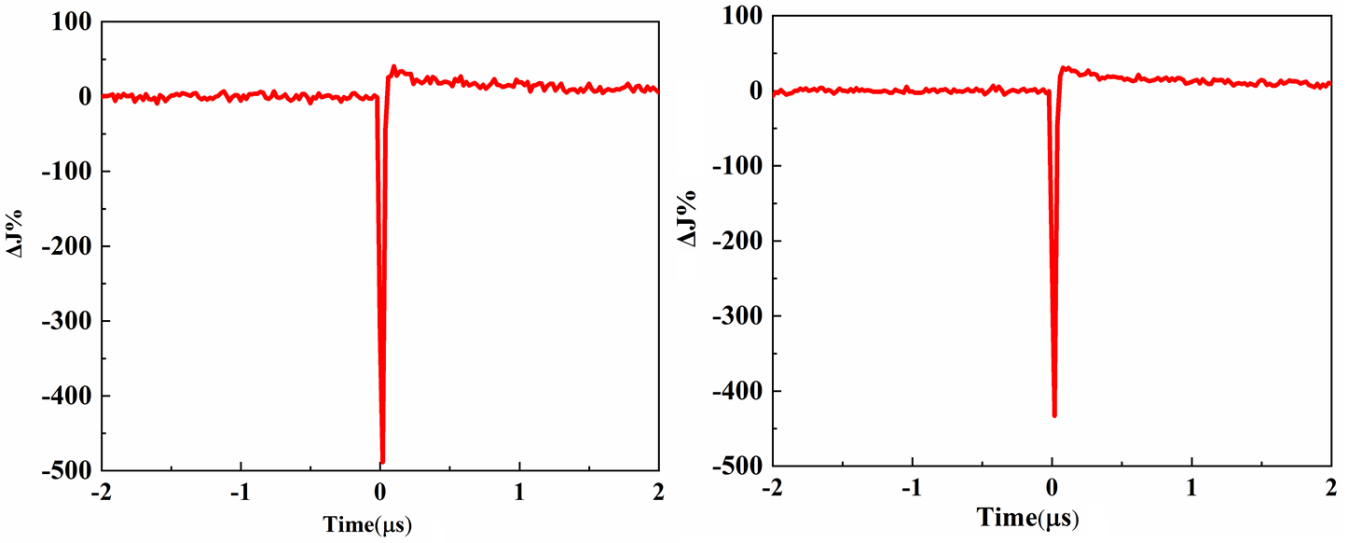


**Figure S5.** Reflectance transition of Nb_2_O_5_/g-C_3_N_4_ after laser excitation at (a) 680 nm (b) 700 nm.

### Calculation for Life-Time

In order to calculate the average life time of decay curve of graphitic carbon nitride, values of time t have taken in column A(X) and values of wavelength I(t) in column B(Y). After this we have added a new column C(Y) by multiplying column A and column B as (Col (A) * Col (B)). Then we plotted a graph between C(Y) and A(X) which is given below:


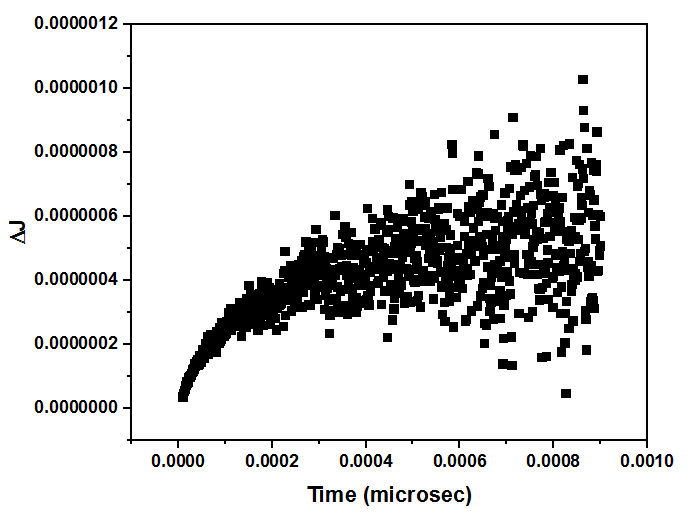


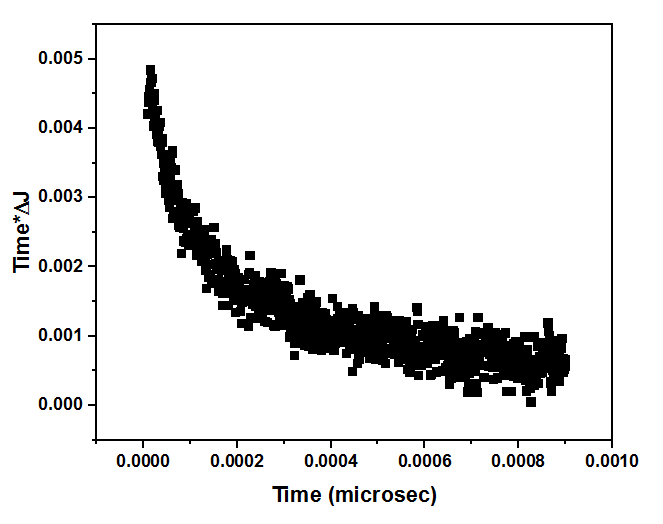
**Figure S6**. Plot of area A_1_ for the calculations of Lifetime of g-C_3_N_4_ in the absence of methanol

**Figure S7**. 10 Plot of area A_2_ for the calculations of Lifetime of g-C_3_N_4_ in the absence of methanol

After that graph have been integrated. By performing all these steps correctly, finally generated the area A_1_ = 3.7933.79384980 x 10^-10^ which is taken as numerator. Then we plotted the graph between B(Y) and A(X) and integrated it by doing the same process as mentioned above. We generated the area A_2_ = 1.198 x 10^-06^ which is taken as denominator of. By dividing area A2 by A1, finally calculated the average life time of decay curve and its value is 31.6518979 μs. Same above process was repeated to calculate the average life time of g-C_3_N_4_/Nb_2_O_5_.
